# Inhibition of Wnt/β-catenin signaling suppresses myofibroblast differentiation of lung resident mesenchymal stem cells and pulmonary fibrosis

**DOI:** 10.1038/s41598-018-28968-9

**Published:** 2018-09-11

**Authors:** Honghui Cao, Cong Wang, Xiang Chen, Jiwei Hou, Zou Xiang, Yi Shen, Xiaodong Han

**Affiliations:** 1 Immunology and Reproduction Biology Laboratory & State Key Laboratory of Analytical Chemistry for Life Science, Medical School, Nanjing University, Nanjing, 210093 China; 2 Jiangsu Key Laboratory of Molecular Medicine, Nanjing University, Nanjing, 210093 China; 3 State Key Laboratory of Natural Medicines and Jiangsu Key Laboratory of Drug Discovery for Metabolic Diseases, Center of New Drug Discovery, China Pharmaceutical University, 24 Tong Jia Xiang, Nanjing, 210009 China; 4 Department of Health Technology and Informatics, Faculty of Health and Social Sciences, The Hong Kong Polytechnic University, Hung Hom, Kowloon, Hong Kong Nanjing, China; 5 Department of Cardiothoracic Surgery, Jinling Hospital, Medical School of Nanjing University, Nanjing, China

## Abstract

An emerging paradigm proposes a crucial role for lung resident mesenchymal stem cells (LR-MSCs) via a fibroblastic transdifferentiation event in the pathogenesis of idiopathic pulmonary fibrosis (IPF). Aberrant activation of Wnt/β-catenin signaling occurs in virtually all fibrotic lung diseases and is relevant to the differentiation of mesenchymal stem cells (MSCs). *In vitro*, by measuring the protein levels of several key components involved in Wnt/β-catenin signaling, we confirmed that this signaling pathway was activated in the myofibroblast differentiation of LR-MSCs. Targeted inhibition of Wnt/β-catenin signaling by a small molecule, ICG-001, dose-dependently impeded the proliferation and transforming growth factor-β1 (TGF-β1)-mediated fibrogenic actions of LR-MSCs. *In vivo*, ICG-001 exerted its lung protective effects after bleomycin treatment through blocking mesenchymal-myofibroblast transition, repressing matrix gene expression, and reducing cell apoptosis. Moreover, delayed administration of ICG-001 attenuated bleomycin-induced lung fibrosis, which may present a promising therapeutic strategy for intervention of IPF. Interestingly, these antifibrotic actions of ICG-001 are operated by a mechanism independent of any disruption of Smad activation. In conclusion, our study demonstrated that Wnt/β-catenin signaling may be an essential mechanism underlying the regulation of myofibroblast differentiation of LR-MSCs and their further participation in the development of pulmonary fibrosis.

## Introduction

Idiopathic pulmonary fibrosis (IPF) is an agnogenic disease characterized by diffuse alveolar inflammation and a fibrotic lung disorder. Its clinical manifestation is dyspnea on exertion, respiratory deterioration, and heart failure, eventually leading to death^1^. The traditional therapeutic drugs empirically employed in the clinic are limited to nonspecific antioxidants, immunosuppressants with undesirable treatment effects and low curative rates^2,3^. Recently, two new drugs, pirfenidone and nintedanib, have shed some light on reducing lung function decline, but they fail to halt or reverse the progression of the disease^4,5^. Lung transplantation is considered to be the only definitive therapy that prolongs survival, although the post-transplantation 5-year survival for IPF patients does not exceed 50%^6^. In this context, it is crucial to explore the pathogenesis of pulmonary fibrosis and find out appropriate therapeutic approaches.

Although the pathogenic mechanism of IPF is unclear, considerable evidence indicates that the disease pathogenesis is initiated through alveolar epithelial cell injury and apoptosis, which results in the accumulation of stem cells responsible for the expansion of the fibroblast and myofibroblast populations in the lungs^7^. Myofibroblasts, a specialized subset of fibroblasts, embody the key features of active fibrosis by their ability to synthesize high levels of extracellular matrix (ECM), making them the key cells that represent the hallmark of IPF^8,9^. Accordingly, defining the sources of these myofibroblasts and abrogating their proliferation and accumulation could serve as a potential treatment strategy for pulmonary fibrosis.

Mesenchymal stem cells (MSCs) are pluripotent stem cells with properties of potential multi-directional differentiation, immune regulation and self-replication^10^. It is believed that tissue resident MSCs exist in most mammalian organs such as the lung, brain, and liver, and their resident tissue niche specifies their function during the development of disease^11,12^. Recent evidence suggests that lung resident mesenchymal stem cells (LR-MSCs) express mRNAs encoding contractile and ECM proteins indicative of a myofibroblast progenitor cell phenotype^13^. The behavior of these LR-MSCs is highly sensitive to the microenvironment to which they are exposed, and they can be driven by local factors to differentiate into myofibroblasts that contribute to lung remodeling at the expense of functional tissue repair^14^. Generally acknowledged as one of the primary causative agents of myofibroblast differentiation, transforming growth factor-1 (TGF-β1) is extensively overexpressed by cells in fibrotic lungs, resulting in the dysregulation of normal lung homeostasis by enhancing ECM accumulation and altering the balance of matrix metalloproteinases (MMPs) and their inhibitors^13,15^.

Wnt/β-catenin signaling is an evolutionarily conserved signal pathway that has been suggested to be crucial in regulating embryonic development and tissue homeostasis. At the cellular level, this signaling controls cell proliferation and motility, generation of cell polarity, and self-renewal of stem cells^16^. We have previously implicated the participation of Wnt/β-catenin signaling in lung repair and pulmonary fibrosis via regulating the differentiation of mouse MSCs into specific cells^17,18^. However, LR-MSCs are located in the interstitial perivascular regions and exhibit the ability to differentiate into cells capable of deleterious remodeling not only in mouse but also in human lungs^19^. To date, it remains unknown whether Wnt/β-catenin signaling works on the transdifferentiation of human LR-MSCs to invasive myofibroblasts. Unraveling this puzzle may have wide-ranging clinical significance.

The hallmark of the activation of Wnt/β-catenin signaling is β-catenin accumulation in the cytoplasm and its translocation into the nucleus, where it binds to T-cell factor (TCF)/lymphocyte enhance factor (LEF). Nuclear β-catenin/TCF subsequently recruits the co-activators cyclic AMP response-element binding protein (CBP) and other components of the basal transcription machinery, to mediate transcription of target genes^20,21^. Along this line, inhibition of the β-catenin-mediated transcriptional complex assembly could be exploited as a potential target to restrain abnormal activation of Wnt/β-catenin signaling in IPF. In this study, we investigated the effect of disrupting Wnt/β-catenin signaling by ICG-001, a peptidomimetic small-molecule inhibitor, on myofibroblast differentiation of mouse LR-MSCs *in vitro*. In the bleomycin-induced mouse pulmonary fibrosis model, the disease pathogenesis is correlated to endogenous LR-MSCs abnormal differentiation and abrogated by ICG-001. More importantly, our finding that ICG-001 also abolished human transforming growth factor (hTGF-β1)-mediated fibrogenic action in human LR-MSCs further suggests that inhibiting Wnt/β-catenin signaling may be an effective strategy for treating fibrotic lung diseases.

## Results

### Human and mouse LR-MSCs demonstrate a typical MSC phenotype and surface marker profile

**Figure 1:**
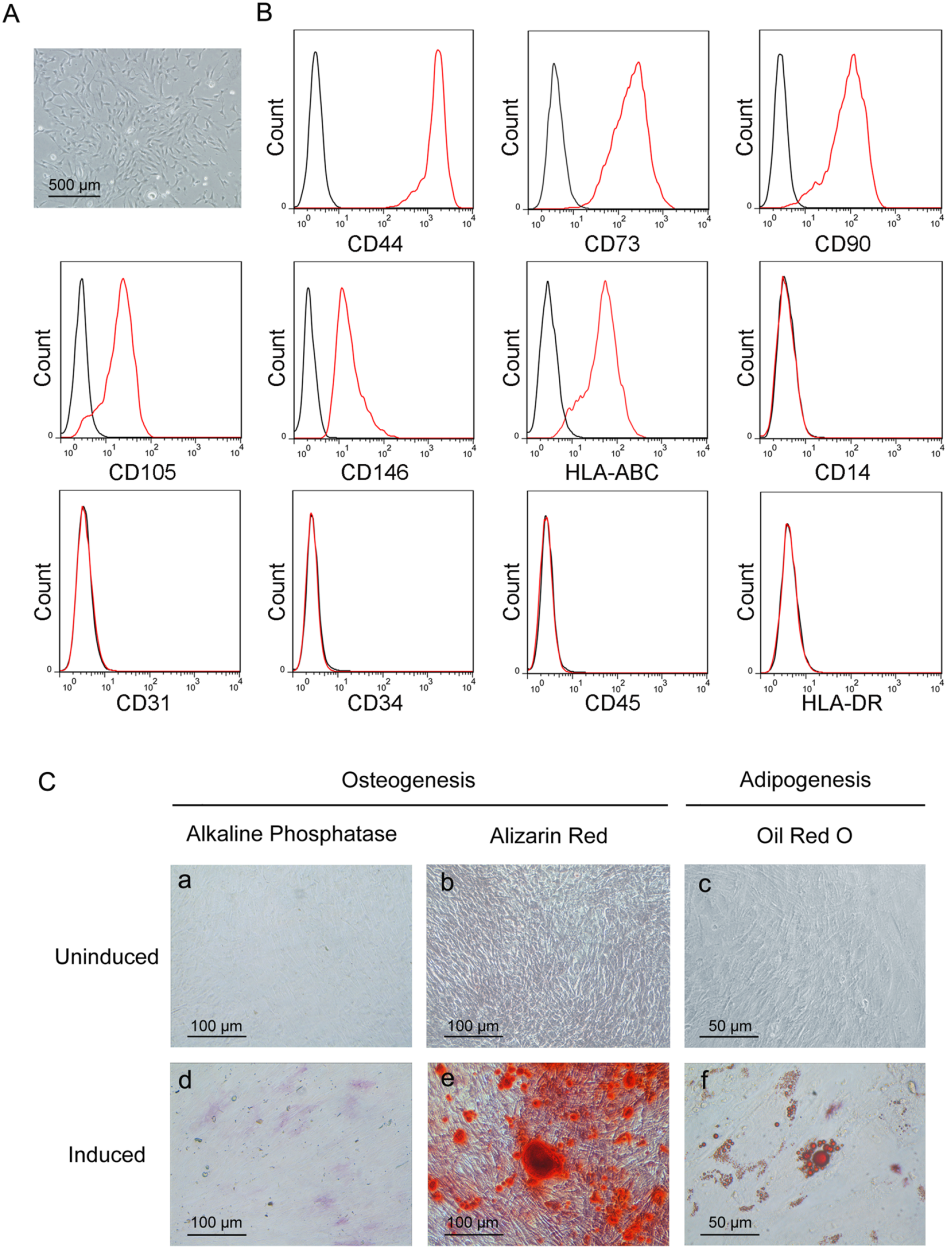
Human lung resident mesenchymal stem cells (LR-MSCs) possess MSC characteristics. (**A**) Morphology of human LR-MSCs was examined by a standard light microscope. (**B**) Cultured human LR-MSCs were harvested, stained with surface marker antibodies and analyzed with flow cytometry. A representative surface marker profile of human LR-MSCs from one individual is shown. (**C**) Human LR-MSCs were cultured in osteogenic differentiation medium and differentiated into osteocytes as evidenced by (d) alkaline phosphatase staining at day 7 and (e) alizarin red staining at day 21. Human LR-MSCs were cultured in adipogenic differentiation media for 21 days and stained with (f) oil red O to determine lipid droplet appearance. (a–c) Control cells were cultured in normal growth medium.

For human LR-MSCs, cell suspensions from lung samples plated in culture medium gave rise to multiple plastic-adherent fibroblast-like cell colonies after approximately 14 days, which were expanded by subsequent trypsinization and several rounds of passaging^22^. These lung-derived adherent cells displayed the morphology of a long spindle, spiral and radial arrangement (Fig. 1A). Immunophenotyping of surface antigens was determined by flow cytometry to characterize this cell population (Fig. 1B). These cells, which are positive for HLA-ABC, expressed markers of mesenchymal progenitors (CD44, CD73, CD90 and CD105) and perivascular cells (CD146). In addition, these cells were negative for hematopoietic (CD45, CD34), endothelial (CD31), and monocyte (CD14) markers and HLA-DR. Together, this pattern of antigen expression is consistent with a typical MSC surface marker profile.

**Figure 2:**
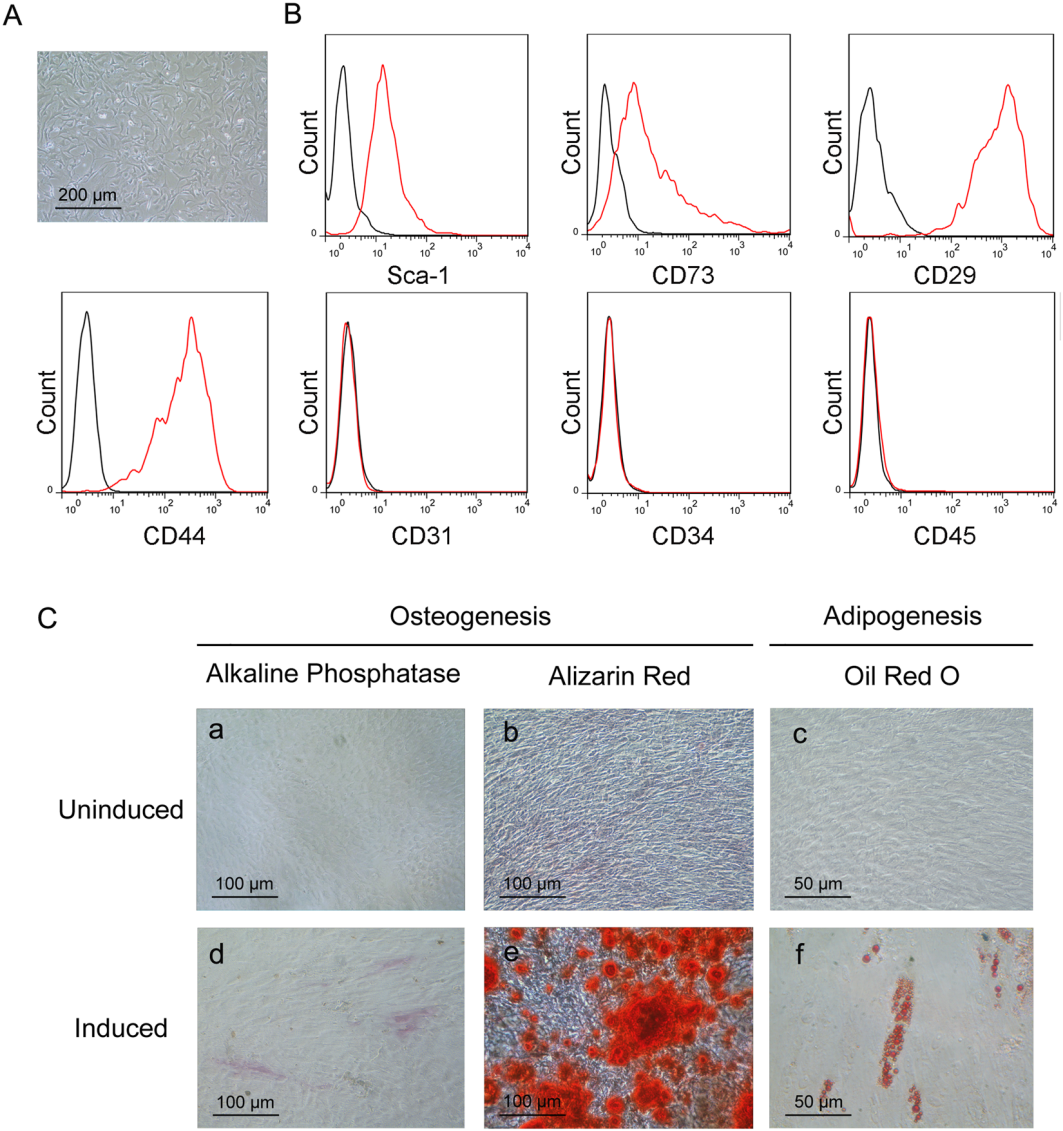
Mouse lung resident mesenchymal stem cells (LR-MSCs) display MSC properties. (**A**) Mouse LR-MSCs morphology after 7 days of culture following isolation by MACS from the mouse lung single-cell suspension was revealed by a standard light microscope. (**B**) The expression of Sca-1, CD73, CD29, CD44, CD31, CD34 and CD45 on mouse LR-MSCs was measured by flow cytometric analysis. The black line indicates negative control and the red line indicates the respective surface marker staining. (**C**) Sorted mouse LR-MSCs were seeded in an appropriate differentiation-induction medium. Osteogenic differentiation of mouse LR-MSCs was demonstrated by (d) alkaline phosphatase staining at day 7 and (e) alizarin red staining at day 21; Adipogenesis was detected by the formation of lipid droplets stained with oil red O at day 21. (a–c) Control cells were cultured in normal growth medium.

Mouse LR-MSCs were sorted from mouse lung cells using MACS^23,24^. Following culture in DMEM for 7 days, these cells were found to adhere to the culture surfaces with a long, thin and stellate morphology resembling bone marrow mesenchymal stem cells (BM-MSCs) (Fig. 2A). The purity of mouse LR-MSCs was confirmed by the surface markers. Indeed, the sorted cells expressed Sca-1, CD73, CD29 and CD44, but not CD31, CD34 or CD45, suggesting that this cell population possessed the main features of MSCs (Fig. 2B). These results indicated that LR-MSCs could be isolated from mouse as well as human lungs, and they displayed similar MSC morphologies and immunophenotypes.

### Human and mouse LR-MSCs possess adipogenic and osteogenic differentiation capacity *in vitro*

Classic MSCs are defined by their capacity for adipogenic and osteogenic differentiation. In assessing this potential in human and mouse LR-MSCs, we found that both cells could be induced to undergo osteogenesis and adipogenesis (Figs 1C and 2C). Osteogenesis in human and mouse LR-MSCs was verified using alkaline phosphatase staining for osteoblasts and alizarin red staining for calcium deposits. Adipogenesis in human and mouse LR-MSCs was characterized by the marked morphological transition from a long spindle shaped cell body to a cobblestone shape cell body and the simultaneous accumulation of lipid droplets which stained with oil red O.

### Wnt/β-catenin signaling is activated during myofibroblast differentiation of human LR-MSCs

**Figure 3:**
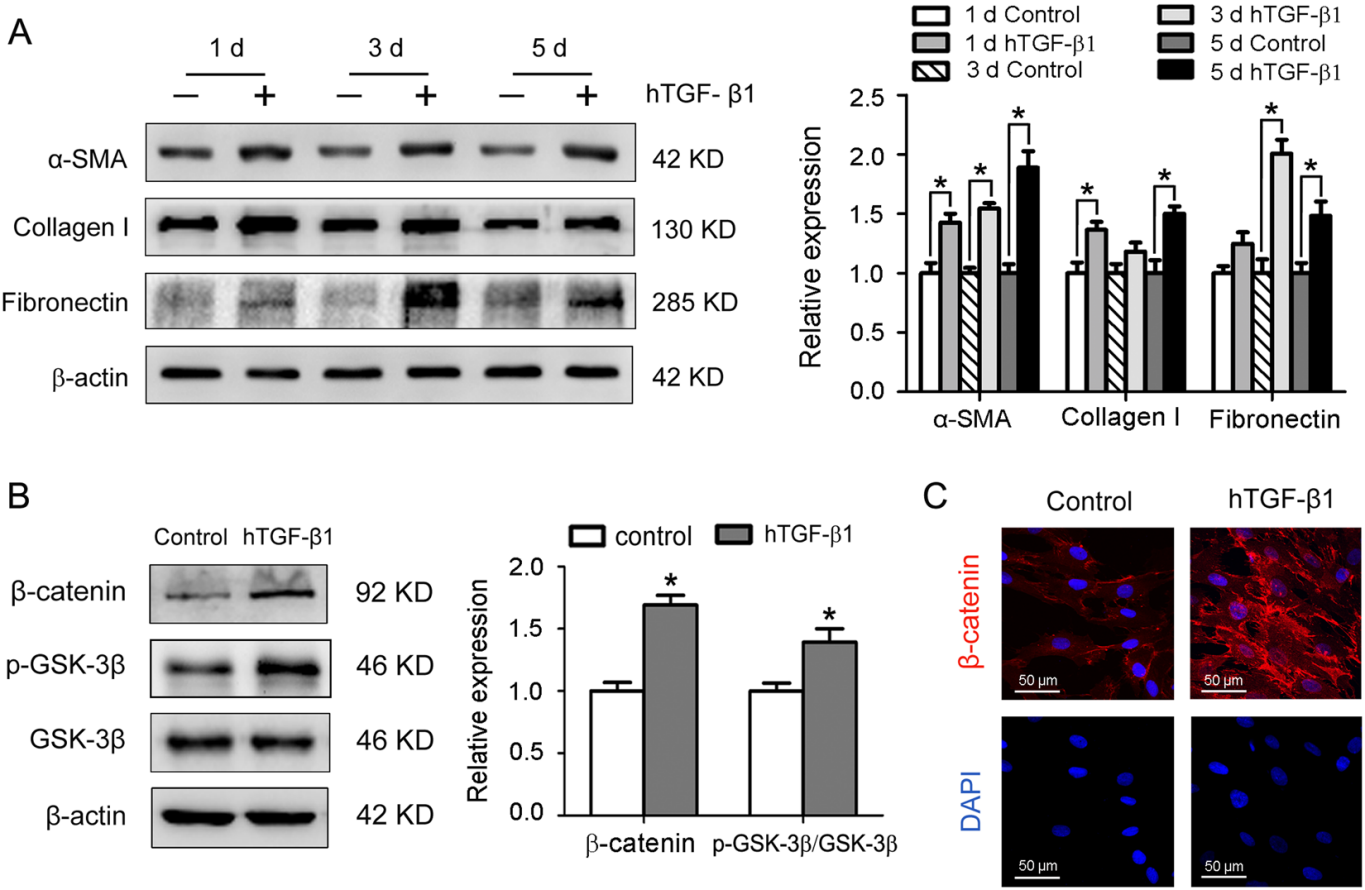
Human transforming growth factor-β1 (hTGF-β1) induces fibrogenic differentiation and Wnt/β-catenin signaling pathway activation in human LR-MSCs. (**A**) The protein expression levels of α-smooth muscle actin (α-SMA), collagen I and fibronectin in human LR-MSCs cultured in medium supplemented with hTGF-β1 (10 ng/ml) for various durations were examined by Western blot. Representative gel electrophoresis bands are shown, and the levels of the proteins were quantified by densitometry and normalized to the expression of β-actin. Densitometry data are shown as mean ± SD. *P < 0.05 versus control. (**B,C**) Human LR-MSCs were incubated with hTGF-β1 (10 ng/ml) for 24 h, followed by measurement of several important nodes of the Wnt/β-catenin signaling pathway. (**B**) The protein expression levels of β-catenin, p-GSK-3β and GSK-3β were determined by Western blot. Representative gel electrophoresis bands are shown, and the levels of the proteins were quantified by densitometry and normalized to the expression of β-actin. Densitometry data are shown as mean ± SD. *P < 0.05 versus control. (**C**) The expression of β-catenin was assessed by immunofluorescence assay. Nuclei were stained with DAPI (blue).

Aberrant activation of Wnt/β-catenin signaling was found in lungs from a subset of IPF patients, indicating the involvement of this pathway in the pathogenesis of pulmonary fibrosis^25^. Following treatment with the profibrotic cytokine hTGF-β1 at 10 ng/ml for 1, 3 and 5 days respectively, human LR-MSCs displayed increased expression of α-smooth muscle actin (α-SMA), collagen I and fibronectin, the major markers of myofibroblasts (Fig. 3A). The expression of Wnt/β-catenin signaling components during the myofibroblast differentiation of human LR-MSCs was also analyzed. Western blot revealed up-regulation of β-catenin and p-GSK-3β in human LR-MSCs after incubation with hTGF-β1 (Fig. 3B). Furthermore, accumulation of β-catenin was confirmed by immunofluorescence staining (Fig. 3C). In conclusion, treatment with hTGF-β1 induced myofibroblast differentiation along with the activation of Wnt/β-catenin signaling in human LR-MSCs.

### Blocking Wnt/β-catenin signaling with ICG-001 inhibits proliferation of human LR-MSCs

**Figure 4:**
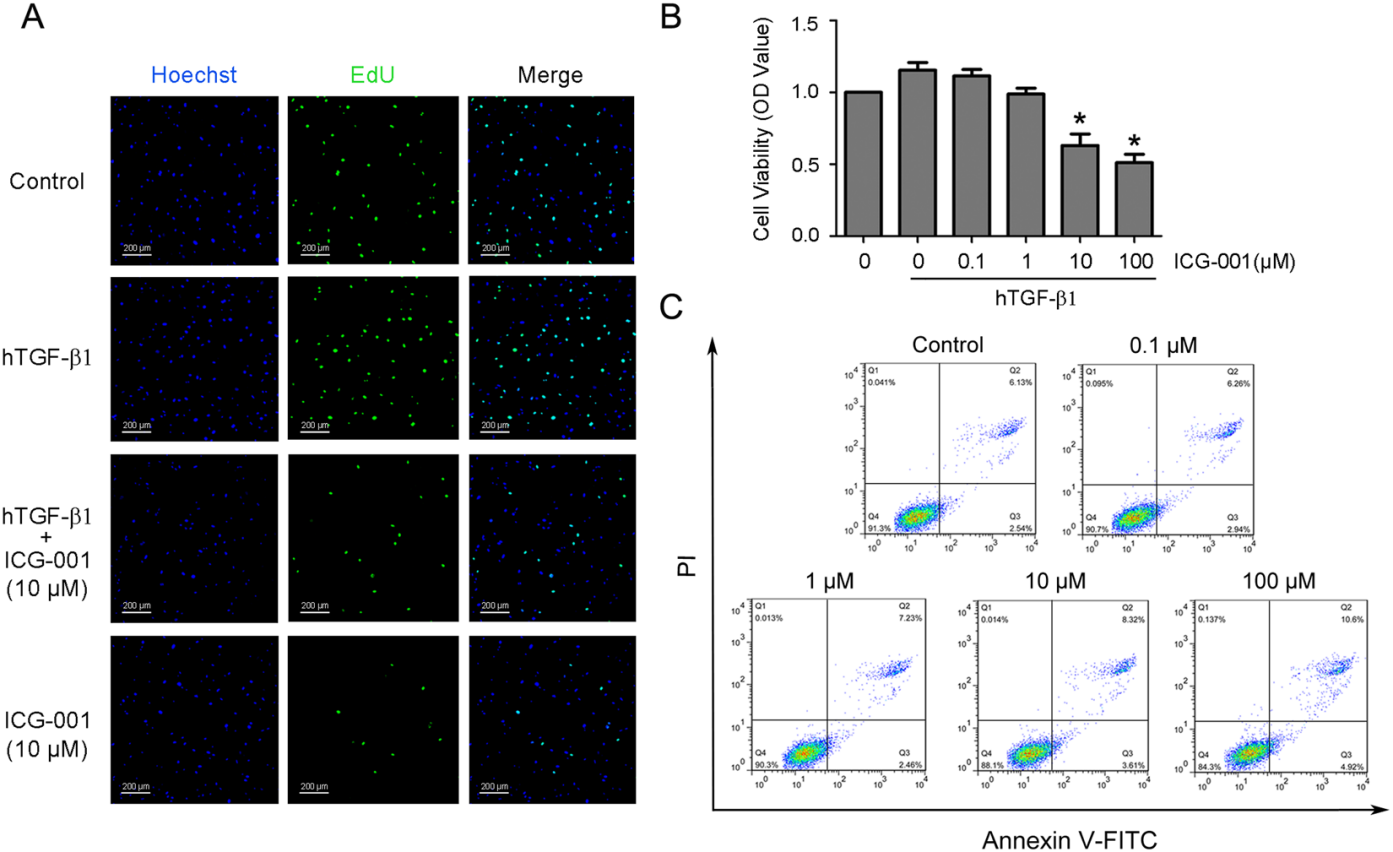
Blocking Wnt/β-catenin signaling with ICG-001 inhibits proliferation of human LR-MSCs. (**A–C**) Human LR-MSCs were treated with ICG-001 at various concentrations in the presence or absence of hTGF-β1 (10 ng/ml) for 24 h. (**A**) Culture medium were pulsed with EdU for 4 h of the 24-h incubation period and subsequently stained with EdU Click-iT reaction mixture (green), together with nuclear staining (blue). (**B**) Cell proliferation was measured with the Cell Counting Kit (CCK-8) assay. *P < 0.05 versus control. (**C**) Cell apoptosis was evaluated by flow cytometry measuring annexin V and propidium iodide (PI) expression. The number in each quadrant represents the percentage of cells in that compartment.

Wnt/β-catenin signaling has been revealed to regulating MSCs proliferation, which is an important mechanism during the progression of IPF. To test this, we applied the peptidomimetic small molecule ICG-001 which selectively disrupts β-catenin/CBP-driven gene transcription^26^. Firstly, viability of human LR-MSCs after treatment with ICG-001 in the presence or absence of hTGF-β1 was assessed by EdU incorporation and CCK-8 assay. Our results demonstrated that the proliferation of human LR-MSCs was promoted by hTGF-β1 while significantly inhibited by ICG-001 at a dose higher than 10 μM (Fig. 4A and B). Furthermore, no remarkably cell death were observed when concentration of ICG-001 was lower than 10 μM (Fig. 4C).

### Blocking Wnt/β-catenin signaling with ICG-001 suppresses myofibroblast differentiation of LR-MSCs by a Smad-independent mechanism

**Figure 5:**
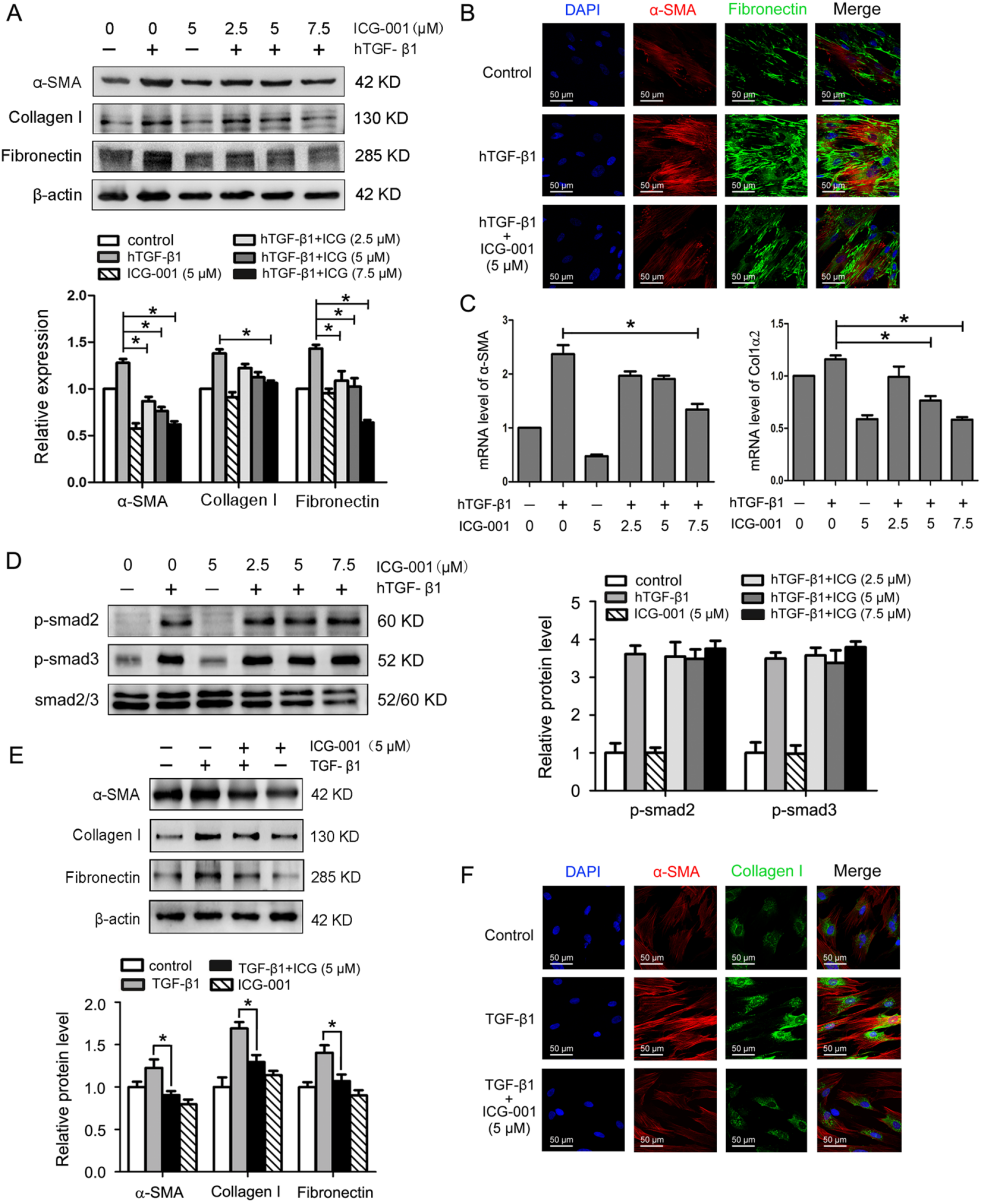
ICG-001 suppresses TGF-β1-mediated myofibroblast differentiation of LR-MSCs by a Smad-independent mechanism. (**A–D**) Human LR-MSCs were treated with ICG-001 at various concentrations in the presence or absence of hTGF-β1 (10 ng/ml) for 24 h. (**A**) The protein levels of α-SMA, collagen I and fibronectin were examined by Western blot. Representative gel electrophoresis bands are shown, and the expression levels of proteins were quantified by densitometry and normalized to the expression of β-actin. Densitometry data are shown as mean ± SD. *P < 0.05 versus hTGF-β1 alone. (**B**) The expression of α-SMA and fibronectin was detected by immunofluorescence assay. Nuclei were stained with DAPI (blue). (**C**) Changes of α-SMA and COL1α2 transcripts were analyzed by Q-PCR. Results are shown as mean ± SD. *P < 0.05 versus hTGF-β1 alone. (**D**) The expression of p-Smad2, p-Smad3 and Smad2/3 was determined using Western blot. Representative gel electrophoresis bands are shown, and the expression levels of proteins were quantified by densitometry and normalized to the expression of smad2/3. Densitometry data are shown as mean ± SD. (**E**,**F**) Mouse LR-MSCs were pretreated with ICG-001 at 5 μM for 1 h, followed by the addition of TGF-β1 into the culture medium to a final concentration of 10 ng/ml and incubation for 24 h. (**E**) The protein levels of α-SMA, collagen I and fibronectin were examined by Western blot. Representative gel electrophoresis bands are shown, and the expression levels of proteins were quantified by densitometry and normalized to the expression of β-actin. Densitometry data are shown as mean ± SD. *P < 0.05 versus TGF-β1 alone. (**F**) The expression of α-SMA and collagen I was assessed by immunofluorescence assay. Nuclei were stained with DAPI (blue).

Given a critical role for Wnt/β-catenin signaling activation during myofibroblast differentiation of human LR-MSCs (Fig. 3), we further examined whether targeted inhibition of this signaling could mitigate fibrogenic responses of both human and mouse LR-MSCs. We measured a number of markers associated with fibrosis and ECM. As presented in Fig. 5A, hTGF-β1 induced protein expression of α-SMA, collagen I and fibronectin in human LR-MSCs, and the expression was effectively inhibited by ICG-001 in a dose-dependent fashion. Immunofluorescence staining confirmed that ICG-001 was able to suppress hTGF-β1-mediated expression of α-SMA and fibronectin in human LR-MSCs (Fig. 5B). Comparable results were obtained when α-SMA and COL1α2 were analyzed at the transcription level (Fig. 5C). Of note, ICG-001 did not affect Smad2 and Smad3 phosphorylation in human LR-MSCs (Fig. 5D), which was in line with a recent report demonstrating that ICG-001 was unable to modulate Smad activation^27^. These results indicated that ICG-001 inhibited hTGF-β1-mediated myofibroblast transition of human LR-MSCs by a mechanism independent of disruption of Smad activation. We also confirmed that ICG-001 suppressed the TGF-β1-induced myofibroblast marker expression in mouse LR-MSCs (Fig. 5E and F), which was consistent with the findings in human LR-MSCs.

### Inhibition of Wnt/β-catenin signaling by ICG-001 attenuates bleomycin-induced pulmonary fibrosis and myofibroblast differentiaion of LR-MSCs *in vivo*

**Figure 6:**
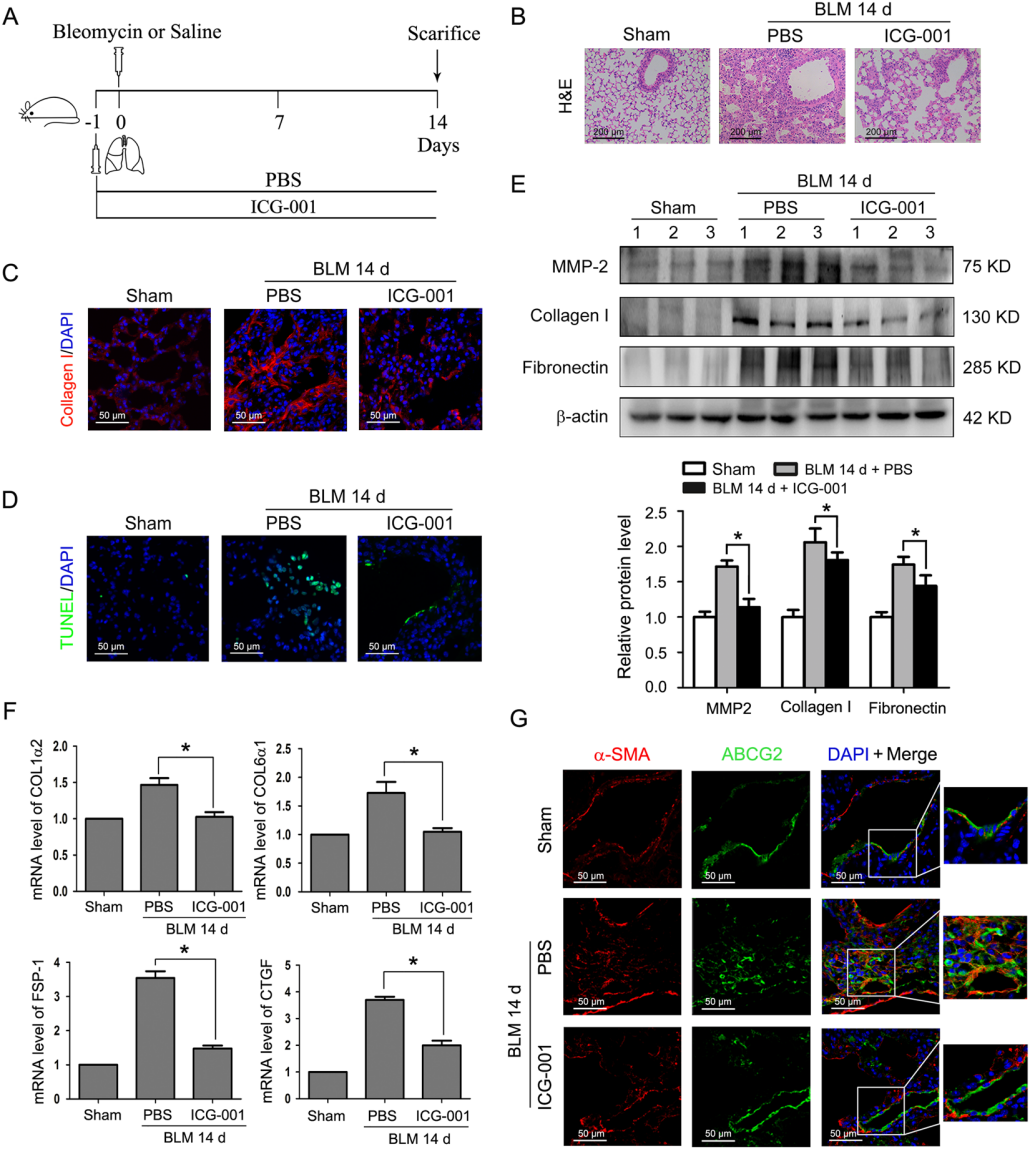
ICG-001 attenuates pulmonary fibrosis and myofibroblast differentiation of LR-MSCs in a bleomycin-induced mouse fibrosis model. (**A**) Schematic presentation of the preventive study design. Mice (n = 6 in each group) started receiving daily intraperitoneal injection of PBS or ICG-001 (5 mg/kg body weight) one day before intratracheal instillation of saline or bleomycin (BLM) (5 mg/kg body weight). Mice were sacrificed on day 14. (**B**) The lung sections from the mice that have undergone different treatments were stained by hematoxylin-eosin (H&E) (**C**) The expression of collagen I in lungs was examined by immunofluorescence assay. Nuclei were stained with DAPI (blue). (**D**) Apoptotic cells of lung tissues were determined by terminal deoxynucleotidyl transferase-mediated dUTP nick end labeling (TUNEL) assay. Nuclei were stained with DAPI (blue) and green dots indicate apoptotic cells. (**E**) The protein levels of matrix metalloproteinase-2 (MMP-2), collagen I and fibronectin in lungs were determined by Western blot. Representative gel electrophoresis bands are shown, and the expression levels of proteins were quantified by densitometry and normalized to the expression of β-actin. Densitometry data are shown as mean ± SD. *P < 0.05 versus BLM 14 d + PBS. (**F**) Changes of the COL1α2, COL6α1, fibroblast-specific protein1 (FSP-1) and connective tissue growth factor (CTGF) transcripts in lungs were measured by Q-PCR. Results are shown as mean ± SD. *P < 0.05 versus BLM 14 d + PBS. (**G**) Myofibroblast differentiation of mouse LR-MSCs *in vivo* was performed using dual immunofluorescence staining to detect ATP-binding cassette transporter subtype G 2 (ABCG2) (green) and α-SMA (red). Nuclei were stained with DAPI (blue).

Having demonstrated the antifibrotic effect of ICG-001 *in vitro*, we next explored whether regulation of Wnt/β-catenin signaling by this small-molecule inhibitor may modulate pulmonary fibrosis *in vivo*. To address this question, ICG-001 was intraperitoneally injected into mice at the dosage of 5 mg/kg one day before bleomycin administration (Fig. 6A). After 14 days, bleomycin injury denuded the alveolar airspaces accompanied by hyperplasia of fibroblasts and extensive collagen deposition, while pretreatment with ICG-001 could improve the lung structure, reduce interstitial fibrosis and decrease cell apoptosis (Fig. 6B–D). Moreover, compared with mice that received bleomycin alone, administration of ICG-001 resulted in profound decreases in the protein expression of MMP-2, collagen I and fibronectin in lung tissues (Fig. 6E). Similarly, the mRNA expression of several well-documented Wnt/β-catenin target and fibrogenic genes was examined by Q-PCR. Bleomycin-induced up-regulation of COL1α2, COL6α1, fibroblast specific protein 1(FSP-1) and connective tissue growth factor (CTGF) was inhibited by ICG-001 (Fig. 6F).

It has been reported that endogenous MSCs mediate pathogenic changes within the lung under certain conditions and act as a critical factor in the development of pulmonary fibrosis^14,28^. Our *in vitro* data clearly demonstrated that LR-MSC populations derived from both mouse and human origins possessed analogous characteristics and are capable of differentiating into myofibroblasts (Figs 1–3 and 5). Hence, we next created a bleomycin-induced mouse fibrosis model to confirm whether mouse LR-MSCs responded to changes in their microenvironment *in vivo*. We assumed that this model may some extent reflect to the behavior of human LR-MSCs in the pulmonary fibrosis patients. The multidrug resistance transporter ATP-binding cassette transporter subtype G 2 (ABCG2) has previously been considered as a marker for LR-MSCs in both murine and human lung tissues, and loss of active ABCG2 expression *in vivo* likely correlates to an altered phenotype and differentiation status of LR-MSCs^12,19^. Here, we found that ICG-001 abolished the transition of LR-MSCs from an ABCG2-expressing stem cell phenotype to a α-SMA-expressing myofibroblast phenotype in bleomycin-treated mice (Fig. 6G). These results suggest that bleomycin induces activation of Wnt/β-catenin signaling in the lung, and inhibition of this signaling pathway by pretreatment with ICG-001 attenuates the mesenchymal-myofibroblast transition *in vivo* and has a protective effect on pulmonary fibrosis.

### Delayed administration of the Wnt/β-catenin signaling inhibitor ICG-001 hinders the progression of pulmonary fibrosis without interfering Smad activation

**Figure 7:**
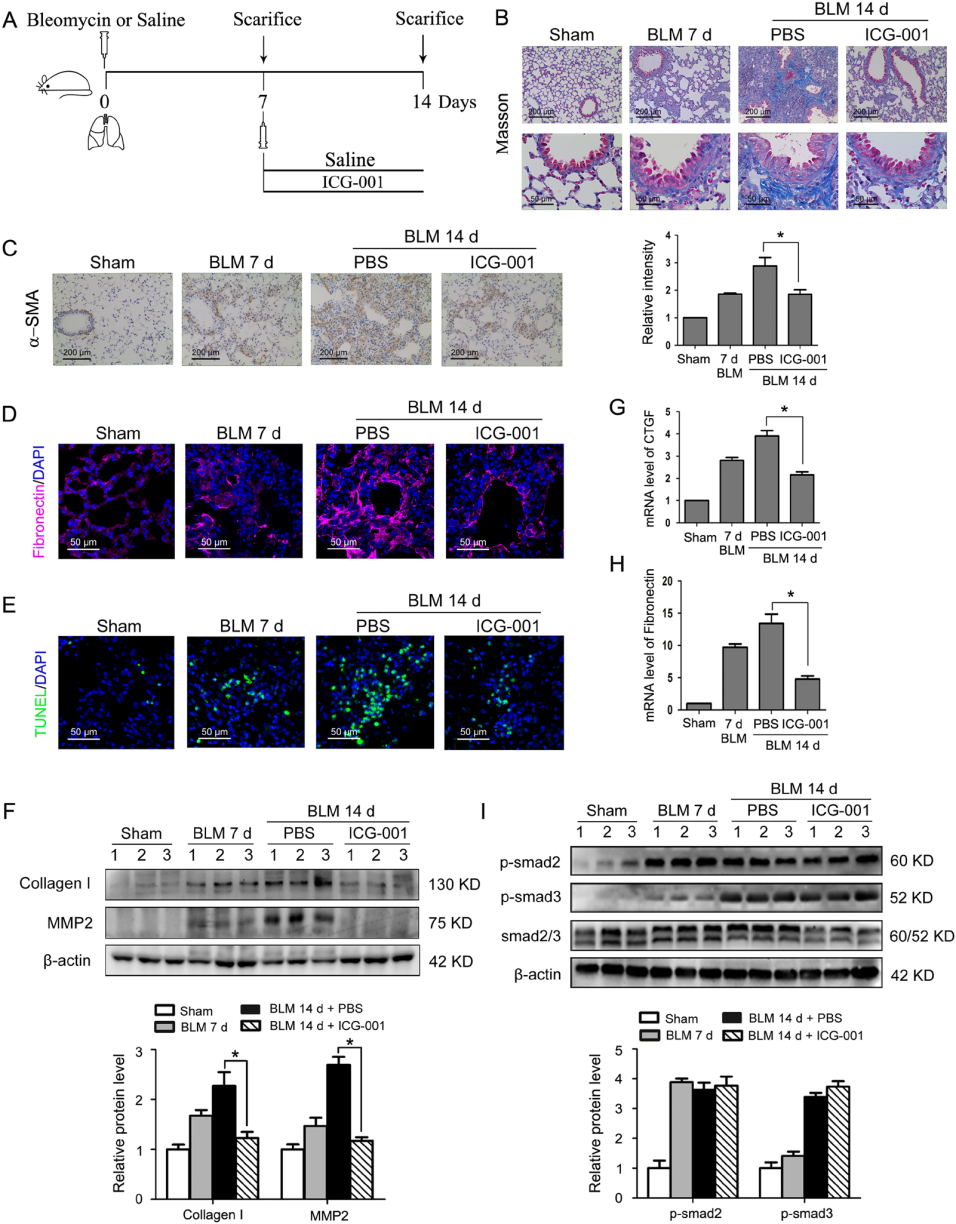
Delayed treatment of ICG-001 halted pulmonary fibrosis progression without interfering Smad activation. (**A**) Schematic presentation of the therapeutic study design. Mice (n = 6 in each group) were daily intraperitoneally injected with PBS or ICG-001 (5 mg/kg body weight) for 7 consecutive days after the administration of saline or bleomycin (BLM) (5 mg/kg body weight). Mice were sacrificed on days 7 or 14. (**B**) Lung sections from various treatment groups were subjected to Masson trichrome staining. (**C**) Immunohistochemistry assay was conducted to measure α-SMA expression in lungs. The positive areas were quantified by densitometry and normalized to sham control. *P < 0.05 versus BLM 14 d + PBS. (**D**) Immunofluorescence assay examined the expression of fibronectin in lungs. Nuclei were stained with DAPI (blue). (**E**) Apoptotic cells of lung tissues were determined by terminal deoxynucleotidyl transferase-mediated dUTP nick end labeling (TUNEL) assay. Nuclei were stained with DAPI (blue) and green dots indicate apoptotic cells. (**F**) The protein expression levels of collagen I and matrix metalloproteinase-2 (MMP-2) in lungs were examined by Western blot. Representative gel electrophoresis bands are shown, and the expression levels of proteins were quantified by densitometry and normalized to the expression of β-actin. Densitometry data are shown as mean ± SD. *P < 0.05 versus BLM 14 d + PBS. (G, H) The mRNA levels of the connective tissue growth factor (CTGF) (**G**) and fibronectin (**H**) in lungs were assessed by Q-PCR. Results are shown as mean ± SD, *P < 0.05 versus BLM 14 d + PBS. (**I**) The protein levels of p-Smad2, p-Smad3 and Smad2/3 were determined by Western blot. Representative gel electrophoresis bands are shown, and the expression levels of proteins were quantified by densitometry and normalized to the expression of smad2/3. Densitometry data are shown as mean ± SD.

To examine the critical therapeutic question of established pulmonary fibrosis, we investigated whether delayed inhibition of Wnt/β-catenin signaling was also able to ameliorate fibrotic lesions in this aggressive model of pulmonary fibrosis (Fig. 7A). As measured by Masson trichrome staining, administration of ICG-001 from day 7 to 14 reduced the deposition of collagen observed in alveolar regions and airways after bleomycin-induced injury (Fig. 7B). Delayed administration of ICG-001 also suppressed α-SMA and fibronectin induction in the interstitium by bleomycin (Fig. 7C and D). Likewise, the extensive apoptosis and expression of collagen I and the ECM cleaving component MMP-2 in the lung tissues was inhibited by late administration of ICG-001 (Fig. 7E and F). Similar findings were observed for CTGF and fibronectin by Q-PCR (Fig. 7G and H). Moreover, ICG-001 exhibited no effect on Smad2 and Smad3 phosphorylation in the lungs of bleomycin-treated mice (Fig. 7I), suggesting that ICG-001 also exerts its antifibrotic action by a mechanism independent of disruption of Smad activation *in vivo*.

## Discussion

IPF, a devastating lung disease with unknown etiology, is featured by progressive deterioration of respiratory functions and poor outcomes. The key histological feature of IPF is the presence of fibroblastic foci in the background of ECM proteins^29^. These fibroblastic foci are revealed as accumulations of fibroblasts and myofibroblasts through epithelial-mesenchymal transition (EMT), the recruitment of circulating fibrocytes, and differentiation of resident mesenchymal cells^13,30,31^. In experimental pulmonary fibrosis, approximately 33% of fibroblasts are derived from EMT, and about 20% fibroblasts originate from the bone marrow progenitor influx^32^. We can speculate that differentiation of local mesenchymal cells or cells from another undisclosed source accounts for the remaining half of the fibroblasts. While the function of EMT as well as fibrocyte traffic from extra-pulmonary sources in lung fibrosis has been well investigated, whether myofibroblast transition of mesenchymal cells especially MSCs facilitates fibrotic disease progression remains to be determined. In this study, we focused on LR-MSCs with respect to their role in the development of pulmonary fibrosis.

Because current treatment approaches in clinical settings are limited and often ineffective, novel therapeutic agents that target key signal pathways involved in the pathogenesis of pulmonary fibrosis to halt disease progression are urgently needed. It is documented that the Wnt/β-catenin signaling pathway is up-regulated and the levels of Wnt isoforms are increased in the lungs of IPF patients and bleomycin-treated mice^33,34^. By isolating human LR-MSCs following incubation with hTGF-β1, we demonstrate that Wnt/β-catenin signaling was activated and was able to drive the differentiation of human LR-MSCs into the myofibroblast lineage, evidenced by the expression of specific molecular markers of myofibroblasts. The Wnt/β-catenin signaling pathway affects cellular functions by regulating β-catenin levels and subcellular localization, and GSK-3β plays a pivotal role in this process. With the phosphorylation occurring at a serine residue in the N-terminus, the GSK-3β activity is inhibited leading to the hypophosphorylation of β-catenin, which allows translocation of this cytoskeletal protein into the nucleus^35^. Subsequent binding of β-catenin to the TCF/LEF family of transcription factors converts them from transcriptional repressors to activators^36^.

Considering a crucial role of Wnt/β-catenin signaling in MSC fate decision and the development of pulmonary fibrosis, we reasoned that inhibiting this signaling might result in the attenuation of mesenchymal-myofibroblast transition. Indeed, blocking Wnt/β-catenin signaling by ICG-001—the specific inhibitor of β-catenin/CBP-driven transcription—impedes fibrogenic action in both human and mouse LR-MSCs. Mechanistically, the action of ICG-001 in disrupting β-catenin-mediated gene transcription is unique, for it selectively prevents β-catenin/CBP complex formation by binding to CBP rather than β-catenin itself^26,37^. In addition, through the EdU incorporation and CCK8 assay of ICG-001, we confirmed that β-catenin/CBP-driven gene transcription is critical for inducing a proliferative state of MSCs.

*In vivo*, our observation that targeted inhibition of Wnt/β-catenin signaling by ICG-001 protects lungs from bleomycin-induced injury is in favor of the concept that hyperactive Wnt/β-catenin signaling is involved in the pathogenesis of IPF. The antifibrotic efficacy of the Wnt/β-catenin inhibitor ICG-001 may be attributed to several potential mechanisms. First and foremost, we observed that LR-MSCs underwent bleomycin-induced phenotypic changes to become α-SMA^+^ myofibroblasts, suggesting that endogenous LR-MSCs failed to effectively repair lung injury and were involved in pulmonary fibrosis instead. As Wnt/β-catenin signaling is activated in the course of mesenchymal-myofibroblast transition *in vitro*, blocking this signaling would inhibit myofibroblast differentiation of LR-MSCs in the fibrotic lungs. This notion is consistent with the findings that ICG-001 repressed LR-MSCs to acquire a fibroblastic phenotype in bleomycin-treated mice. Secondly, bleomycin alone may not be sufficient to induce myofibroblast transition in LR-MSCs; down modulators of injury and fibrosis, such as MMPs and TGF-β1, clearly play a role in the maladaptive differentiation of LR-MSCs^19,38^. Elevated concentrations of MMP-2 were reported in the fibroblastic foci of IPF, and dysregulation of MMP activity accounts for functional alterations and tissue damage^39^. MMP-mediated degradation of ECM has also been implicated as a physiological mechanism for the release and activation of TGF-β, which leads to enlarged ECM deposition in fibrosis^15,38^. Consequently, inhibiting Wnt/β-catenin signaling by ICG-001 can elicit lung protective effects by repressing the expression of MMP-2, a member of the MMP family involved in the deregulation of synthesis and degradation of ECM proteins. Thirdly, ICG-001 may exert its antifibrotic action through preventing the expression of FSP-1 and fibronectin, two Wnt/β-catenin signaling target genes associated with EMT or ECM deposition^40,41^. Moreover, induction of fibrotic cytokine CTGF paralleled the progression of fibrogenesis. As a mediator of TGF-β1 expression and an enhancer of ECM deposition, the presence of CTGF promotes formation of myofibroblasts through transdifferentiation of other cells including epithelial cells, fibrocytes and MSCs^42^. It is therefore conceivable that blocking Wnt/β-catenin signaling by ICG-001 can inhibit the CTGF induction and further reduce ECM production.

The pathological processes involved in bleomycin-induced rodent pulmonary fibrosis can be represented by biphasic responses, i.e. the early inflammatory reaction phase (7 days) and the subsequent fibrosis phase (apparently on day 14)^43^. To avoid disrupting inflammation and be more relevant to clinical settings, we injected ICG-001 intraperitoneally 7 days after bleomycin administration. It is known that bleomycin recognize and noncovalently bind to specific sequences in DNA in the presence of ferrous ion and then to perform chemistry on a specific deoxynucleoside residue that can ultimately lead to strand scission. Inhibition of Wnt/β-catenin signaling in advance, the apoptosis at the single-cell level was reduced in the lungs of bleomycin-treated mice. More importantly, late administration of ICG-001 also suppressed the fibrogenic action and DNA strands breaks of cells induced by bleomycin, for it interrupted the continuous activation of Wnt/β-catenin signaling. Besides, these therapeutic actions are operated by a mechanism independent of any disruption of Smad activation.

In summary, the present work, supported by multiple lines of *in vitro* and *in vivo* evidence, unraveled a mechanism by which Wnt/β-catenin signaling regulates the differentiation of LR-MSCs into myofibroblasts. Targeted inhibition of Wnt/β-catenin signaling by ICG-001, a small molecule, could attenuate the capability of LR-MSCs for acquiring fibroblastic properties. In addition, ICG-001 was capable of ameliorating fibrotic lesions through blocking the expression of a number of fibrogenic genes and cytokines in a bleomycin-induced lung injury model. We also identified that the antifibrotic effect of ICG-001 did not involve interference with Smad signaling. Despite the fact that more investigations are needed to thoroughly dissect the mechanisms, our study fills a gap in connecting LR-MSCs with their role in the development of pulmonary fibrosis via the Wnt/β-catenin signaling pathway, which may have an implication for the treatment of fibrotic lung diseases.

## Materials and Methods

### Isolation of human and mouse LR-MSCs

The animal experiments were conducted in accordance with the Guide for the Care and Use of Laboratory Animals (The Ministry of Science and Technology of China, 2006) and all experimental protocols have been approved by the Animal Care and Use Committee of Nanjing University. Lung samples from patients who provided informed and written consent following permission from the ethical committee of Nanjing University were used for preparation of human LR-MSCs, and all methods were performed in accordance with the guidelines and regulations.

Human LR-MSCs were isolated and cultured as previously reported^22^. Briefly, fresh human specimens were obtained from the distal lung of 10 patients undergoing pneumonectomy for pneumothorax. These individuals are generally young, healthy, non-smokers who require the resection of a congenital sub-pleural bleb to prevent pneumothorax recurrence. The minced lung parenchyma tissue was placed in an enzyme mixture containing 0.2% collagenase I (Sigma-Aldrich, St. Louis, Mo), 2.4 U/ml dispase (Sigma) for 1 h at 37 °C with shaking. This suspension was filtered followed by centrifugation. Next, lung cells were seeded in DMEM containing 10% fetal bovine serum, 4% L-glutamine, 1% nonessential amino acids, and 1% penicillin and streptomycin, and maintained in a humidified atmosphere of 95% air, 5% CO_2_ at 37 °C. Single separated fibroblastoid colonies were identified at a mean interval of 2 weeks after initial plating. A homogeneous population of MSCs was obtained from all colonies of an individual patient.

C57BL/6 mice aged 4-6 weeks were purchased from the Medical School of Yangzhou University (Yangzhou, China). Mouse LR-MSCs were isolated and cultured as previously reported by us and others^18,44^. In brief, mouse lung parenchyma tissues were digested after cutting with a razor blade. The digested material was filtered and depleted of red blood cells by Ammonium-Chloride-Potassium (ACK) lysis buffer. Cells were resuspended in PBS and stained for Sca-1 and CD45 followed by sorting using the magnetic-activated cell sorting (MACS) (Miltenyi Biotec, Bergisch Gladbach, Germany). Freshly isolated LR-MSCs were cultured with DMEM containing 10% fetal bovine serum, 4% L-glutamine, 1% nonessential amino acids, and 1% penicillin and streptomycin, and maintained in a humidified atmosphere of 95% air, 5% CO_2_ at 37 °C. Both human and mouse LR-MSCs were passaged 1:2 using 0.25% trypsin when they reached 70–90% confluence.

### Flow cytometric analysis

To determine the cell-surface antigen phenotypes, cells were incubated with fluorescent antibodies at 37 °C for 1 h in the dark followed by two washes with PBS. The antibodies used for human LR-MSCs included: FITC–conjugated anti-CD34, CD45; PE-conjugated anti-CD90, CD105, CD146 (all from Miltenyi Biotec), CD14, CD31, CD73, HLA-ABC, HLA-DR (all from Becton Dickinson, San Jose, CA) and CD44 (eBioscience, San Diego, CA). The antibodies used for mouse LR-MSCs included: FITC-conjugated anti-Ly-6A/E (Sca-1); PE-conjugated anti-CD34, CD29, CD73, CD31 (all from Becton Dickinson), CD44 and CD45 (eBioscience). Cell apoptosis was analyzed by an annexin V-FITC and propidium iodide (PI) staining kit (Vazyme, Nanjing, China) according to manufacturer’s instructions. Human LR-MSCs were transplanted to six-well culture plates at a density of 1 × 10^6^ cells/ml and were cultured for 12 h followed by treatment with various concentrations of ICG-001 (0, 0.1 μM, 1 μM, 10 μM and 100 μM) for 24 h. Cells were collected after centrifugation at 300 g for 5 min. Next, cells were washed twice with cold PBS and were resuspended in 500 μl binding buffer. Each cell sample was then stained with 5 μl Annexin V-FITC and 5 μl PI and incubated in the dark at 25 °C for 15 min. Flow cytometry was performed on a FACS CaliburTM flow cytometer (Becton Dickinson) and the data were analyzed with FlowJo software (Tree star, Ashland, Oregon).

### Osteogenic Differentiation

Human and mouse LR-MSCs were seeded at a density of 4 × 10^3^ cells/cm^2^ on 12-well plates in standard growth medium until they reached 80% confluence. The cells were then incubated in osteogenic induction medium, which is basic medium (DMEM low glucose with 10% FBS) supplemented with 100 nM dexamethasone, 50 μg/ml ascorbic acid, and 10 mM β-glycerolphosphate (Invitrogen, Carlsbad, CA). For alkaline phosphatase staining, after 7-day osteogenic culture, cells were fixed in 4% formaldehyde for 5 min, washed twice using 20 mM Tris buffer and stained by alkaline phosphatase staining solution (Millipore, Billerica, MA) for 20 min at room temperature, then cells were washed with buffer. For alizarin red staining, after 21-day osteogenic culture, cells were fixed in 70% ice ethanol, washed twice using ddH_2_O and dyed for 30 min with alizarin red solution (Millipore) at room temperature, then rinsed with ddH_2_O for four times and the images were taken.

### Adipogenic Differentiation

Human and mouse LR-MSCs were seeded at a density of 4 × 10^3^ cells/cm^2^ on 12-well plates in standard growth medium until they reached 80% confluence. The medium was then replaced with adipogenic induction medium, which includes basic medium (DMEM low glucose with 10% FBS), 0.1 μM dexamethasone, 0.5 mM isobutylmethylxanthine, 50 μM indomethacin, and 5 μg/ml of insulin (Invitrogen). The cells were cultured for another 21 days. Then, cells were fixed with 4% formaldehyde for 40 min at room temperature, following washed twice in PBS and stained with oil red O Solution (Millipore). After a 50-min incubation at room temperature, cells were washed twice using ddH_2_O and the images were taken.

### LR-MSC treatment

Human LR-MSCs were treated with or without hTGF-β1 (Cell Signaling Technology, Beverly, MA) at 10 ng/ml for indicated periods of time, and then harvested for analysis of the myofibroblast markers and the key components of Wnt/β-catenin signaling. A small-molecule inhibitor, ICG-001, which specifically disrupts β-catenin signaling in a CBP-dependent fashion has been reported in previous literature^26^. Both human and mouse LR-MSCs were pretreated with ICG-001 (Selleckchem, Houston, TX) for 1 h, followed by incubation with 10 ng/ml hTGF-β1 or TGF-β1 (PeproTech, Rocky Hill, NJ) for 24 h. The cells were collected for Q-PCR, Western blot analysis, and immunofluorescence staining.

### EdU incorporation assay

Cell proliferation was assessed by 5-ethynyl-2′-deoxyuridine (EdU) incorporation using the Click-iT EdU Alexa Fluor 488 cell proliferation assay kit (Invitrogen). Human LR-MSCs were grown on glass coverslips in DMEM containing 10% FBS, 4% L-glutamine, 1% nonessential amino acids, and 1% penicillin and streptomycin. Then, human LR-MSCs were treated with 10 μM ICG-001 for 24 h in the presence or absence of hTGF-β1 (10 ng/ml). The EdU solution was added to the medium during the last 4 h of the 24-h incubation period. After labeling, cells were fixed with 4% paraformaldehyde for 15 min at room temperature. Then cells were washed with 3% BSA in PBS and were incubated with 0.5% Triton X-100 in PBS for 20 min at room temperature. EdU-labeled cells were visualized using Click-iT reaction cocktails (Invitrogen). Cells were incubated with Hoechst 33342 to visualize nuclei, then analyzed using a confocal fluorescence microscope (FV10i; Olympus, Tokyo, Japan).

### CCK-8 assay

Cell Counting Kit-8 (CCK-8, Dojindo Laboratories, Kumamoto, Japan), which measures cell viability, is based on the conversion of an orange-colored product from water-soluble tetrazolium salt (WST-8) by dehydrogenases in live cells. Human LR-MSCs were treated with various concentrations of ICG-001 (0, 0.1 μM, 1 μM, 10 μM and 100 μM) in the presence or absence of hTGF-β1 (10 ng/ml) in 96-well plates for 24 h followed by the CCK-8 assay according to the instructions from the manufacturer.

### RNA extraction and quantitative real-time polymerase chain reaction (Q-PCR)

Total RNA was extracted from cultured cells or lung tissues using Column Animal RNAout according to the manufacturer’s instructions. The purity of RNA was determined with a spectrophotometer (Hoefer, Holiston, MA). An Easyscript first-strand cDNA synthesis super mix kit (Vazyme) was used for reverse transcription polymerase chain reaction (RT-PCR). Q-PCR was conducted using the SYBR Green Q-PCR kit (Roche, Germany) on a ViiA 7 Q-PCR system (Applied Biosystems, Waltham, MA). Each sample was run in triplicate, and PCR reactions without the addition of the template were used as blank controls. The relative quantification of the expression of the target genes was measured using glyseraldehyde-3-phosphate dehydrogenase (GAPDH) mRNA as an internal control. The sequences of primer pairs used in this assay are listed in Table S1.

### Western blot

Proteins were purified from either cells or lung tissues. Western blot analysis was performed as previously described^35^. Proteins were separated using 10% SDS-PAGE and electrophoretically transferred to polyvinylidene fluoride (PVDF) membranes. The primary antibodies employed were: rabbit anti-glycogen synthase kinase-3β (GSK-3β), rabbit anti-p-GSK-3β, rabbit anti-smad2/3 (Cell Signaling Technology), rabbit anti-β-catenin, rabbit anti-fibronectin, mouse anti-matrix metalloproteinase-2 (MMP-2), mouse anti-β-actin, rabbit anti-p-smad2, rabbit anti-p-smad3 (Abcam, Cambridge, MA), rabbit anti-collagen I, and mouse anti-α-SMA. Species-matched horseradish peroxidase-conjugated IgG (Boster, Wuhan, China) was used as the secondary antibody. The chromogenic signal intensity was detected using an Odyssey Scanning System (LI-COR, Lincoln, NE) and quantified using image J software (NIH, Bethesda, MD).

### Bleomycin-induced mouse pulmonary fibrosis model

C57BL/6 mice weighing approximately 20 to 22 g were maintained under standard conditions with free access to water and laboratory rodent food. After anesthesia with pentobarbital sodium (3 mg/kg), mice received a single, slow intratracheal injection of 5 mg/kg bleomycin (Nippon Kayaku Co, Tokyo, Japan) dissolved in 50 μl of saline with MicroSprayer (Penn-Century, Wyndmoor, PA). Sham control mice received 50 μl of saline instead. In the preventive study (Fig. 6A), PBS or ICG-001 was administered into mice by daily intraperitoneal injections at the dosage of 5 mg/kg body weight. One day later, mice received bleomycin or saline intratracheally. Mice were sacrificed 14 days after bleomycin instillation. In the therapeutic study (Fig. 7A), mice received daily treatment of ICG-001 (5 mg/kg body weight) or PBS by intraperitoneal injection starting on day 7 for a 7-day treatment period after administration of bleomycin or saline intratracheally on day 0. Mice were sacrificed on day 7 or day14.

### Histopathology

The mouse lungs were fixed in 4% neutral phosphate-buffered paraformaldehyde overnight, dehydrated, transparentized and embedded in paraffin before sectioning into 5 μm-thick slices. The sections were stained with hematoxylin-eosin (H&E) for structured observation, or with Masson trichrome stain for detection of collagen deposits, or were subjected to TUNEL assay and immunohistochemistry/immunofluorescence analyses.

### Immunohistochemistry and TUNEL assay

Five μm-thick slides were deparaffinized with xylene before rehydration using an ethanol gradient. Next, quenching of endogenous peroxidase activity was achieved by incubation with 3% H_2_O_2_. After blocking with 3% BSA, the sections were incubated with mouse anti-α-SMA overnight at 4 °C. The secondary antibodies incubated were horseradish peroxidase-conjugated goat anti-mouse IgG (Boster). The DAB Substrate System (DAKO) was used to reveal the immunohistochemical staining.

TUNEL assay as described by the manufacturer (Roche Applied Science, Mannheim, Germany), and the nuclei were stained with 4′ 6-diamidino-2-phenylindole (DAPI) (Sigma). The images were captured using a laser scanning confocal fluorescence microscope (Olympus).

### Immunofluorescence staining

The immunofluorescence analysis of LR-MSCs or lung tissues were performed as described previously^27^. The following primary antibodies were employed: rabbit anti-β-catenin, mouse anti-α-SMA, rabbit anti-collagen I, rabbit anti-fibronectin, and rabbit anti-ATP binding cassette transporter subfamily G member 2 (ABCG2) (Abcam). Alexa Fluor 488-conjugated goat anti-rabbit antibody or Alexa Fluor 594-conjugated goat anti-mouse antibody (Invitrogen) was used as the secondary antibody. Nuclei were stained with DAPI (Sigma). The images were captured using a confocal fluorescence microscope (Olympus).

### Statistical analysis

Experimental results were expressed as means ± standard deviation. Differences were analyzed for significance (P < 0.05) by one-way ANOVA using SPSS for windows version 11.0 (SPSS, Chicago, IL).

## Electronic supplementary material

Supplementary Information
