# Supplementary material for: Inhibition of Wnt/β-catenin signaling suppresses myofibroblast differentiation of lung resident mesenchymal stem cells and pulmonary fibrosis
Source: Sci Rep. 2018 Sep 11;8:13644. doi: 10.1038/s41598-018-28968-9 (PMC6134002; doi:10.1038/s41598-018-28968-9)
Supplement: Supplementary file 1 — Supplementary Information [file 41598_2018_28968_MOESM1_ESM.pdf]

Inhibition of Wnt/β-catenin signaling suppresses myofibroblast differentiation of lung resident mesenchymal stem cells and pulmonary fibrosis.

Honghui Cao[1,2], Cong Wang[3], Xiang Chen[1,2], Jiwei Hou[1,2], Zou Xiang[4], Yi Shen[5*] & Xiaodong Han[1,2*]

1 Immunology and Reproduction Biology Laboratory & State Key Laboratory of Analytical Chemistry for Life Science, Medical School, Nanjing University, Nanjing, 210093, China.

2 Jiangsu Key Laboratory of Molecular Medicine, Nanjing University, Nanjing, 210093, China

3 State Key Laboratory of Natural Medicines and Jiangsu Key Laboratory of Drug Discovery for Metabolic Diseases, Center of New Drug Discovery, China Pharmaceutical University, 24 Tong Jia Xiang, Nanjing, 210009, China

4 Department of Health Technology and Informatics, Faculty of Health and Social Sciences, The Hong Kong Polytechnic University, Hung Hom, Kowloon, Hong Kong, China.

5 Department of Cardiothoracic Surgery, Jinling Hospital, Medical School of Nanjing University.

Mailing address: Immunology and Reproductive Biology Laboratory, Medical College of Nanjing University, Hankou Road 22, Nanjing, 210093, China

Department of Cardiothoracic Surgery, Jinling Hospital, Medical School of Nanjing University, 305 East Zhongshan Road, Nanjing, 210002, China.

***Corresponding author:** Yi Shen and Xiaodong Han

E-mail address: yishen305@126.com and hanxd@nju.edu.cn

Tel: +86-25-83686497

Fax: +86-25-83686497

Supplementary File

Table 1. Sequence of mouse and human PCR primers used for Q-PCR.

Genes	Forward primer (5’ to 3’)	Reverse primer (5’ to 3’)
Mouse		
Fibronectin	TGTGACAACTGCCGTAGACC	GACCAACTGTCACCATTGAGG
FSP-1	GATGAGCAACTTGGACAGCA	ATGTGCGAAGAAGCCAGAGT
CTGF	GTGTCTTCGGTGGGTCGGTGTA	TGGCTCGCATCATAGTTGGGT
COL1α2	GTCCTAGTCGATGGCTGCTC	CAATGTCCAGAGGTGCAATG
COL6α1	TGCCAAGGACTTCATCATCA	CTCCATTCCCCAGTGTGTTT
GAPDH	AACTTTGGCATTGTGGAAGG	ACACATTGGGGTAGGAACA
Human		
α-SMA	ACCCACAATGTCCCCATCTA	GACAATCTCACGCTCAGCAG
COL1α2	GGCCCTCAAGGTTTCCAAGG	CACCCTGTGGTCCAACAACTC
GAPDH	AAATGCTGTTGAGCACGATG	CTGTGCTGGATATCGCCTTT
